# Redox-Guided Epigenetic Signaling in Cancer: miRNA–DNMT Feedback Loops as Epigenetic Memory Modulates

**DOI:** 10.3390/antiox15030295

**Published:** 2026-02-27

**Authors:** Moon Nyeo Park

**Affiliations:** College of Korean Medicine, Kyung Hee University, 1-5 Hoegidong, Dongdaemun-gu, Seoul 02447, Republic of Korea; mnpark@khu.ac.kr

**Keywords:** epigenetic memory, DNA methyltransferase (DNMT), microRNA (miRNA), DNMT–miRNA feedback loop, bistable regulatory circuits, cancer cell plasticity, epigenetic reprogramming, herbal medicine, phytochemicals, translational epigenetic therapy, redox signaling

## Abstract

Epigenetic dysregulation is a central driver of cancer progression, therapeutic resistance, and phenotypic plasticity. Among epigenetic mechanisms, microRNAs (miRNAs) and DNA methyltransferases (DNMTs) engage in reciprocal regulatory interactions that extend beyond transient gene control. Emerging evidence indicates that DNMT–miRNA feedback loops function as epigenetic memory units, stabilizing malignant cell states and enabling durable phenotypic inheritance even after removal of initiating stimuli under conditions shaped by persistent redox and stress signaling cues. In this review, we synthesize mechanistic, computational, and translational studies demonstrating how double-negative DNMT–miRNA feedback architectures generate bistable regulatory circuits that lock cancer cells into epithelial–mesenchymal transition, stem-like, and therapy-resistant states through redox-sensitive regulatory thresholds rather than static epigenetic alterations. This framework provides a unifying explanation for why transient environmental or therapeutic cues can induce long-lasting epigenetic reprogramming and why conventional single-target epigenetic inhibitors often fail to achieve durable clinical responses. Building on this concept, we propose that herbal medicines and plant-derived phytochemicals act as epigenetic reset signals capable of destabilizing pathological epigenetic attractor states encoded by DNMT–miRNA memory circuits by modulating intracellular redox balance and redox-responsive signaling pathways. Owing to their multi-component and systems-level regulatory properties, herbal interventions modulate miRNA expression, DNMT activity, and upstream stress-responsive pathways in a coordinated manner, facilitating transitions from memory-dominated states toward renewed epigenetic plasticity. We further discuss the translational implications of combining miRNA-based therapies with herbal medicine as a strategy for epigenetic reprogramming rather than transient suppression within a redox-guided therapeutic framework. Finally, we address key challenges and clinical feasibility considerations, including delivery, heterogeneity, and safety, and outline future directions for biomarker-guided and systems-informed epigenetic therapies that incorporate redox state as a functional determinant of epigenetic responsiveness. By reframing DNMT–miRNA interactions through the lens of epigenetic memory, this review highlights miRNA–herbal combination strategies as a forward-looking approach for overcoming therapeutic resistance and achieving durable reprogramming in cancer through selective manipulation of redox-sensitive epigenetic signaling circuits.

## 1. Conceptual Framework

### 1.1. miRNA as an Epigenetic Regulator in Cancer

MicroRNAs (miRNAs) function not only as post-transcriptional regulators of gene expression but as active components of epigenetic regulatory machinery that shapes chromatin states and transcriptional programs in cancer cells [1,2]. Beyond their canonical role in messenger RNAs, they regulate key epigenetic enzymes, including DNA methyltransferases (DNMTs), histone deacetylases (HDACs), and polycomb group proteins such as enhancer of zeste homolog 2 (EZH2), thereby reshaping epigenetic landscapes at both global and locus-specific levels [2,3]. Dysregulated miRNAs participate in bidirectional feedback loops with epigenetic modifiers, forming self-reinforcing circuits that stabilize oncogenic or tumor-suppressive transcriptional states [4,5]. In this context, tumor-suppressive miRNAs are frequently silenced through promoter CpG island hypermethylation and repressive histone marks, whereas oncogenic miRNAs may be epigenetically activated to sustain malignant phenotypes [1,4]. Such reciprocal regulation underlines the concept of “epi-miRNAs,” a subclass of miRNAs that both regulate and are controlled by epigenetic mechanisms, thereby occupying a central position within epigenetic control networks [5]. Mechanistically, miRNAs can directly target epigenetic enzymes at the post-transcriptional level, thereby modulating chromatin accessibility and transcriptional output. For example, multiple tumor-suppressive miRNAs inhibit DNMT1, DNMT3A, or DNMT3B expression, leading to DNA demethylation and reactivation of silenced tumor suppressor genes [2,6]. Similarly, miRNAs targeting histone-modifying enzymes, including HDACs and EZH2, contribute to dynamic remodeling of histone acetylation and methylation marks associated with epithelial–mesenchymal transition (EMT), stemness, and therapy resistance [3,7]. Conversely, epigenetic enzymes regulate miRNA expression through coordinated DNA methylation and histone modification of miRNA gene promoters. DNMT1- and EZH2-mediated silencing of tumor-suppressive miRNAs, including members of the miR-200 family, miR-31, miR-142-3p, and miR-484, has been observed across multiple cancer types and is consistently associated with invasion, metastasis, and poor clinical outcomes [1,8,9]. These findings highlight miRNA loci as critical epigenetic targets whose deregulation contributes to cancer progression. Importantly, miRNA–epigenetic interactions are not linear but are embedded within complex regulatory networks involving transcription factors, chromatin modifiers, and signaling pathways. Transcriptional regulators such as SOX4 can cooperate with EZH2 and HDACs to repress miRNA expression, while the suppressed miRNAs in turn target components of the same epigenetic complexes, creating tightly coupled regulatory circuits that reinforce malignant cell states [8]. Such multilayered regulation enables cancer cells to achieve phenotypic plasticity and adapt to environmental and therapeutic stress [4,7].

Importantly, accumulating evidence indicates that miRNA–epigenetic feedback networks are highly sensitive to cellular redox states, positioning reactive oxygen species (ROS) as upstream signaling determinants rather than mere by-products of metabolic stress. Oxidative stress has been shown to modulate the transcription, processing, and stability of specific miRNAs, thereby reshaping epigenetic landscapes in a context-dependent manner. For instance, ROS-driven signaling alters miRNA transcription, processing, and stability through redox-sensitive transcription factors such as NF-κB and NRF2, thereby reshaping epigenetic landscapes in a context-dependent manner [10,11]. In parallel, oxidative stress further influences the activity and expression of DNA methyltransferases and demethylases, linking redox imbalance to dynamic changes in DNA methylation patterns and transcriptional memory [12]. Notably, experimental evidence demonstrates that ROS modulation can alter miRNA-controlled antioxidant networks, exemplified by miR-21-mediated disruption of SOD2- and NRF2-dependent ROS homeostasis, thereby reinforcing redox–epigenetic feedback loops that stabilize stress-adaptive cellular states [13]. These findings support a model in which miRNA–epigenetic circuits function as redox-responsive regulatory modules, integrating oxidative signals into persistent epigenetic configurations that govern cancer cell plasticity, therapy resistance, and disease progression. Collectively, these studies establish miRNAs as central regulators of epigenetic homeostasis in cancer, functioning at the interface between RNA-based regulation and chromatin remodeling. Understanding the molecular logic of miRNA-epigenetic feedback networks provides a conceptual foundation for therapeutic strategies aimed at restoring epigenetic balance through targeted miRNA modulation or epigenetic enzymes intervention, with translational implications for biomarker development and precision oncology [2,5,6].

### 1.2. Redox-Signaling as an Upstream Modulator of miRNA–Epigenetic Circuits

ROS function as bona fide signaling molecules that dynamically shape epigenetic regulation in cancer. Spatially confined and moderated ROS pulses act as upstream modulators of chromatin states and transcriptional programs, whereas sustained or excessive oxidative stress promotes genomic instability and cell death. Within this spectrum, redox signaling provides a critical regulatory interface through which environmental, metabolic, and therapeutic stresses are translated into stable epigenetic adaptations. ROS-sensitive signaling pathways, including hypoxia-inducible factor-1α (HIF-1α), nuclear factor erythroid 2–related factor 2 (NRF2), and metabolic stress sensors, directly influence DNA methyltransferases, histone-modifying enzymes, and non-coding RNA networks, thereby coupling redox dynamics to long-term epigenetic memory [14,15]. At the molecular level, redox signaling modulates epigenetic enzymes through both transcriptional and post-translational mechanisms. Oxidative stress alters the availability of metabolic cofactors, such as α-ketoglutarate, NAD^+^, and acetyl-CoA, thereby modulating the activity of histone demethylases, sirtuins, and chromatin acetyltransferases. The identification of a nonclassical nuclear tricarboxylic acid (TCA) cycle illustrates direct metabolic–epigenetic coupling, in which redox-regulated metabolites fine-tune chromatin accessibility and transcriptional output. These findings establish redox states as integral components of epigenetic regulation rather than external modifiers [15]. Redox signaling intersects with miRNA regulation to establish self-reinforcing feedback loops. ROS-responsive transcription factors regulate the expression of specific miRNAs, while redox-sensitive miRNAs, in turn, target components of antioxidant systems, metabolic pathways, and epigenetic machinery. For example, perturbations in glutathione homeostasis and cystine transport have been shown to remodel chromatin states through SIRT6-dependent histone acetylation, linking redox balance to epigenetic reprogramming. Such mechanisms exemplify how redox–miRNA–epigenetic circuits function as adaptive regulatory modules that stabilize malignant phenotypes under stress [16]. Beyond cancer, redox–epigenetic coupling has been observed across diverse biological contexts, including stress adaptation and aging, reinforcing the concept that ROS-mediated chromatin remodeling represents a conserved regulatory strategy. In pathological settings, however, cancer cells exploit this system to lock in pro-survival, therapy-resistant states. Consequently, redox signaling should be viewed not as a parallel pathway but as an upstream regulatory layer that licenses epigenetic plasticity and miRNA-mediated gene regulation. This perspective provides a mechanistic foundation for redox-guided therapeutic interventions aimed at destabilizing maladaptive epigenetic memory in cancer. The therapeutic relevance of redox–miRNA–epigenetic coupling lies in redox thresholds and signaling amplitude-dependent effects, whereby transient and moderate redox signaling induces reversible epigenetic remodeling, whereas sustained or oxidative stress drives irreversible DNA methylation and chromatin locking associated with therapy resistance and disease progression [17,18]. From a therapeutic standpoint, these observations indicate that precise modulation of redox signaling—rather than global antioxidant suppression—can reprogram miRNA–epigenetic circuits toward plastic, drug-responsive states, thereby reopening therapeutic windows otherwise closed by fixed epigenetic memory [19,20,21].

### 1.3. Redox-Guided Therapeutic Reprogramming Through miRNA and Natural Products

Effective therapeutic reprogramming in cancer requires an integrated understanding of redox signaling, miRNA regulation, and epigenetic plasticity rather than isolated targeting of single molecular nodes [11,15,22]. ROS function as context-dependent signaling entities that dynamically shape transcriptional and epigenetic states through redox-sensitive kinases, transcription factors, and chromatin modifiers [14,23]. Within this framework, miRNAs function as signal integrators that convert transient redox fluctuations into sustained regulatory outputs by modulating epigenetic enzymes, stress-response pathways, and cell fate determinants [24,25]. Redox imbalance influences miRNA biogenesis and function at multiple levels. Oxidative stress alters the activity of key miRNA-processing components, including DROSHA and DICER, while redox-sensitive transcription factors such as NF-κB, HIF-1α, and NRF2 directly regulate miRNA transcription [26,27]. In turn, specific miRNAs target antioxidant systems, mitochondrial regulators, and epigenetic enzymes, forming closed-loop redox–miRNA–epigenetic circuits that stabilize stress-adaptive phenotypes [28]. These circuits contribute to therapy resistance, metabolic rewiring, and phenotypic plasticity by reinforcing epigenetic memory states, underscoring their relevance as therapeutic targets [16,29]. Natural products and multi-component herbal formulations are uniquely positioned to intervene in such complex regulatory networks through redox-guided modulation. Unlike single-target synthetic agents, many phytochemicals exhibit redox-modulating properties that fine-tune ROS signaling rather than indiscriminately suppressing oxidative processes [30,31]. Accumulating studies demonstrate that natural compounds can restore redox balance while simultaneously reprogramming miRNA expression profiles associated with oncogenic signaling, EMT, and stemness [22,32]. Through this dual action, natural products can destabilize maladaptive epigenetic memory encoded by redox-responsive miRNA networks [33]. Redox-guided therapeutic strategies emphasize reprogramming rather than cytotoxic elimination. By shifting the redox tone of cancer cells toward a non-permissive state, malignancy-associated miRNA expression patterns linked to malignancy can be destabilized, enabling partial restoration of tumor-suppressive transcriptional programs [16,34]. This concept is consistent with evidence that excessive antioxidant therapy may be ineffective or even detrimental, whereas precision modulation of redox signaling yields more predictable biological outcomes [14,15]. In this context, miRNA signatures responsive to redox perturbation offer promising biomarkers for patients’ stratification and therapeutic monitoring [27,29]. Collectively, redox-guided miRNA reprogramming provides a conceptual framework that integrates oxidative signaling, epigenetic regulation, and natural product pharmacology into a coherent therapeutic paradigm [26,31]. Such an approach supports the development of system-level interventions capable of overcoming resistance mechanisms and achieving durable modulation of cancer cell states, with implications extending beyond oncology to aging-associated diseases and longevity-related redox disorders [22,30].

## 2. DNA Methylation–miRNA Interactions

### 2.1. Herbal Medicine-Derived miRNAs Targeting DNMTs

Aberrant DNA methylation is a hallmark of cancer progression, encompassing promoter hypermethylation of tumor suppressor genes and global hypomethylation that collectively reshape oncogenic transcriptional programs. DNA methyltransferases (DNMTs), particularly DNMT1, DNMT3A, and DNMT3B, play central roles in establishing and maintaining these epigenetic abnormalities, representing critical regulatory nodes within cancer-associated epigenetic networks [1]. microRNAs (miRNAs) act as endogenous modulators of DNMT expression, thereby influencing DNA methylation dynamics and cancer cell fate decisions [3]. Herbal medicine-derived bioactive compounds function as potent regulators of DNMT-targeting miRNAs, offering a mechanistically distinct mode of epigenetic modulation compared to direct enzymatic inhibition. Multiple phytochemicals restore the expression of tumor-suppressive miRNAs that directly target DNMT transcripts, resulting in partial or global DNA demethylation and reactivation of silenced tumor suppressor genes [35]. This miRNA-mediated regulation introduces an additional layer of epigenetic control, whereby natural compounds indirectly remodel the cancer methylome through RNA-based mechanisms rather than direct DNMT blockade. Among DNMT-regulating miRNAs, the miR-29 family represents one of the most extensively characterized examples. miR-29a, miR-29b, and miR-29c directly target DNMT3A and DNMT3B, and their downregulation in cancer is closely associated with aberrant DNA methylation and malignant progression [4]. Notably, several herbal-derived compounds upregulate miR-29 expression, leading to decreased DNMT levels and demethylation of tumor suppressor gene promoters, thereby suppressing cancer cell proliferation and invasiveness [36]. In addition to the miR-29 family, miRNAs such as miR-148a, miR-152, and miR-101 regulate DNMT expression and are frequently silenced by promoter hypermethylation in cancer cells, forming self-reinforcing epigenetic loops [5]. Herbal interventions disrupt these loops by restoring miRNA expression, which in turn downregulates DNMT1 and attenuates maintenance methylation during cell division [37]. This dual action—reactivating miRNA expression while suppressing DNMT activity—highlights the capacity of herbal medicines to reprogram epigenetic memory rather than transiently inhibit enzymatic function. Mechanistically, DNMT-targeting miRNAs induced by herbal compounds contribute to broad transcriptional reprogramming beyond individual gene loci. Reduced DNMT activity leads to chromatin relaxation, increased accessibility of transcription factor binding sites, and re-expression of genes involved in cell cycle arrest, apoptosis, and differentiation [38]. These effects are often accompanied by reduced EMT and diminished metastatic potential, directly linking miRNA–DNMT regulation to clinically relevant cancer phenotypes [7]. From a translational perspective, miRNA-mediated DNMT modulation by herbal medicines offers several conceptual advantages. Unlike synthetic DNMT inhibitors, which can induce global hypomethylation and off-target toxicity, herbal compounds exert context-dependent and network-level regulation, preferentially restoring tumor-suppressive miRNA programs in cancer cells while sparing normal tissues [39]. This property aligns with emerging paradigms of epigenetic precision therapy, in which reversible and adaptable modulation of the epigenome is favored over irreversible enzymatic inhibition. Collectively, these findings position herbal medicine–derived miRNAs as critical intermediaries linking natural compounds to DNA methylation control in cancer. By targeting DNMTs through endogenous RNA regulatory circuits, herbal interventions exemplify a systems-level epigenetic strategy that integrates miRNA biology, chromatin remodeling, and phenotypic reprogramming, providing a strong mechanistic rationale for their inclusion in epigenetic and RNA-based cancer therapeutics [35].

### 2.2. Conceptual Framework: DNMT–miRNA Feedback Loops as Epigenetic Memory Units in Cancer

DNMT–miRNA feedback loops constitute epigenetic regulatory architectures that extend beyond transient gene regulation and function as memory-encoding units capable of stabilizing cancer cell states over time [1,2]. Unlike linear signaling pathways, in which gene expression rapidly adapts to upstream fluctuations, these feedback loops integrate RNA-based regulation with DNA methylation to transform short-lived stimuli into durable epigenetic states that persist across cell divisions [3]. At the core of this phenomenon is reciprocal repression between miRNAs and DNA methyltransferases. Tumor-suppressive miRNAs frequently target DNMT1, DNMT3A, or DNMT3B transcripts, thereby reducing DNA methylation activity, whereas DNMT-mediated promoter hypermethylation silences transcription of the same miRNAs [4]. This double-negative feedback configuration generates bistable regulatory circuits with toggle-switch-like behavior, enabling cells to occupy one of two relatively stable epigenetic attractor states rather than continuously fluctuating between them [5,25]. Once a critical threshold is crossed, the feedback loop becomes self-sustaining through noise-resistant reinforcement, effectively locking transcriptional programs into place [25,40]. This bistability provides a mechanistic explanation for why cancer cells frequently fail to revert to their original phenotypic states even after the removal of initiating stimuli. Transient environmental cues such as inflammatory signaling, hypoxia, or chemotherapy exposure can trigger initial shifts in miRNA or DNMT expression; however, once DNMT–miRNA feedback loops are engaged, these changes are reinforced through heritable DNA methylation patterns that persist independently of the original signal [6]. Consequently, epigenetic reprogramming transitions from a plastic and reversible phase to a memory-dominated state marked by phenotypic inertia, as bistable feedback topology suppresses stochastic reversal [25,41]. Experimental evidence across multiple cancer types supports this model. In gastric cancer, reciprocal repression between miR-200c and DNMT3A establishes a stable epigenetic circuit that maintains EMT states and invasive behavior, even after upstream EMT-inducing signals have subsided [7]. Similarly, in cisplatin-resistant lung cancer, DNMT1-mediated silencing of miR-30a/c and miR-30a/c–dependent suppression of DNMT1 form a self-reinforcing loop that preserves drug-resistant phenotypes despite drug withdrawal [42]. These findings underscore that DNMT–miRNA feedback loops encode cellular history rather than merely reflecting current signaling conditions. DNMT–miRNA memory circuits are rarely isolated. They are frequently embedded within broader regulatory networks involving transcription factors, histone modifiers, and chromatin-associated proteins that collectively enhance epigenetic robustness. Transcriptional regulators such as PAX5 or SOX family members cooperate with DNMTs to silence miRNA loci, while the suppressed miRNAs simultaneously target DNMTs and associated epigenetic cofactors, reinforcing multilayered repression [8]. This network integration reduces stochastic noise and further stabilizes epigenetic memory states [43]. From a systems-level perspective, DNMT–miRNA feedback loops resemble bistable switches whose topology enables history-dependent state fixation and long-term epigenetic memory [5,25]. Mathematical and computational modeling studies demonstrate that DNMT-miRNA feedback circuits maintain stable epigenetic states over extended time scales, even in the presence of fluctuating upstream inputs, by virtue of their bistable topology and noise-buffering properties, thereby providing a formal framework for understanding state-based epigenetic inheritance in cancer [9,25]. These models align with experimental observations that cancer-associated DNA methylation patterns are mitotically stable yet reversible under appropriate reprogramming conditions [3]. Within this conceptual framework, miRNA-based therapies can be reinterpreted not merely as gene-silencing interventions but as epigenetic reprogramming strategies. By targeting key nodes within DNMT–miRNA feedback loops, miRNA therapeutics have the potential to destabilize entrenched epigenetic memory states and restore phenotypic plasticity [2,25]. This capacity to unlock fixed cancer cell identities distinguishes miRNA-based approaches from conventional cytotoxic or signaling-targeted therapies and provides a mechanistic rationale for their long-term therapeutic impact [44]. Collectively, DNMT–miRNA feedback loops function as epigenetic memory units that explain why cancer cell states, once established, are resistant to spontaneous reversal, why transient environments or therapeutic stimuli induce durable phenotypic transition, and why RNA-based interventions can achieve genuine epigenetic reprogramming rather than transient gene suppression. Framing these interactions as memory-encoding regulatory circuits provides a unifying conceptual lens through which RNA-based epigenetic regulation, cancer plasticity, and therapy resistance can be systematically understood and therapeutically targeted [1,3,5,25]. Experimental and computational evidence indicates that DNMT–miRNA feedback loops operate as bistable epigenetic memory units across diverse cancer types, stabilizing malignant phenotypes through reciprocal repression mechanisms. Representative examples of such DNMT–miRNA circuits, their molecular configurations, and associated epigenetic and phenotypic outcomes are summarized in Table 1, highlighting their role in maintaining epithelial–mesenchymal transition, stemness, and therapy-resistant states.

### 2.3. Distinction from Conventional Epi-miRNA Models

Conventional epi-miRNA models describe reciprocal regulatory interactions between DNA methyltransferases (DNMTs) and microRNAs within relatively stable chromatin landscapes. These frameworks are largely grounded in epigenetic memory paradigms, in which DNA methylation and histone modifications sustain transcriptional states across cell divisions [47,48]. In such models, miRNAs function either as downstream repressors of DNMT expression or as targets of promoter methylation, forming feedback loops that reinforce transcriptional stability and lineage commitment. However, these classical frameworks insufficiently integrate redox dynamics into epigenetic regulation. Redox and metabolic reprogramming are tightly coupled processes in cancer and stress adaptation, operating as coordinated signaling networks rather than isolated molecular interactions [49]. ROS function as signal modulators whose amplitude and spatial distribution critically determine transcriptional outcomes [50], and alterations in intracellular redox status have been shown to directly influence gene expression programs and differentiation trajectories [51]. In contrast to static epi-miRNA feedback loops, we propose that the DNMT–miRNA axis functions as a redox-conditioned epigenetic circuit. In this model, oxidative flux does not merely induce secondary methylation changes but actively gates DNMT activity, miRNA biogenesis, and chromatin accessibility in a context-dependent manner. Redox-sensitive transcriptional regulators such as NRF2 directly modulate DNMT expression and components of the miRNA processing machinery [52], embedding oxidative signaling into the epigenetic control layer. Accordingly, the DNMT–miRNA interaction should be interpreted not solely as a memory-stabilizing structure but as a dynamic regulatory module responsive to intracellular redox oscillations and metabolic stress. This distinction repositions the DNMT–miRNA framework from a static epigenetic feedback paradigm toward an integrated redox-epigenetic regulatory architecture. ROS act as upstream regulators of the epigenetic landscape by directly modulating DNA methylation and demethylation machinery. ROS influence DNMT expression and catalytic activity and can interfere with CpG methylation fidelity through oxidative DNA lesions such as 8-oxo-deoxyguanosine, thereby reshaping promoter methylation patterns in a redox-dependent manner [53]. In parallel, redox imbalance affects Fe^2+^/α-ketoglutarate-dependent TET dioxygenases, altering 5-methylcytosine oxidation dynamics and contributing to aberrant methylation turnover under oxidative stress conditions [54]. Importantly, ROS-induced DNMT1 recruitment to CpG islands within tumor-suppressive microRNA loci has been shown to drive promoter hypermethylation and miRNA silencing, establishing a direct mechanistic axis linking oxidative stress to epigenetically mediated microRNA repression [54].

### 2.4. Herbal Medicine as an Epigenetic Reset Signal

**Figure 1:**
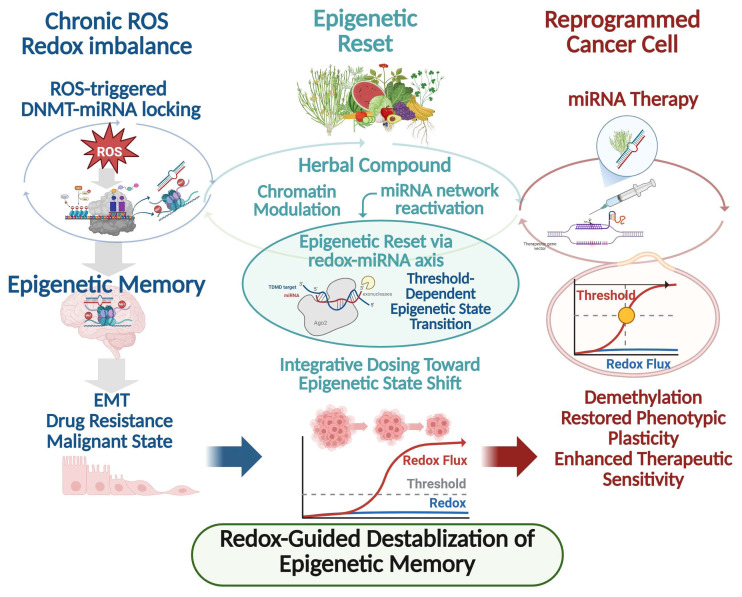
Redox-guided destabilization of DNMT–miRNA epigenetic memory and therapeutic reprogramming in cancer. Chronic redox imbalance characterized by sustained reactive oxygen species (ROS) signaling promotes the stabilization of DNMT–miRNA feedback loops, leading to ROS-triggered locking of bistable epigenetic circuits. These DNMT–miRNA regulatory architectures function as epigenetic memory units that enforce persistent DNA hypermethylation, suppression of tumor-suppressive miRNAs, and stabilization of malignant phenotypes, including EMT, drug resistance, and cancer stem cell–like states. Herbal medicines and plant-derived phytochemicals act as epigenetic reset signals by coordinately modulating chromatin accessibility, DNMT activity, intracellular redox balance, and reactivation of silenced miRNA networks. This systems-level perturbation destabilizes pathological epigenetic memory states and reopens a transitional window of epigenetic plasticity. In parallel, miRNA-based therapeutic interventions directly disrupt DNMT–miRNA feedback circuits, facilitating DNA demethylation, restoration of phenotypic plasticity, and enhanced therapeutic sensitivity. Collectively, redox-guided epigenetic resetting enables reprogramming of malignant cancer cells toward less aggressive and more treatment-responsive states. Threshold-dependent transitions and bistable switching behavior are conceptually illustrated to reflect redox-conditioned DNMT–miRNA circuit dynamics and epigenetic memory stabilization. Abbreviations: DNMT, DNA methyltransferase; miRNA, microRNA; ROS, reactive oxygen species; EMT, epithelial–mesenchymal transition; CSC, cancer stem cell.

Conventional epigenetic therapies, including DNMT or histone deacetylase inhibitors, are largely designed to suppress discrete enzymatic activities and often exert transient effects that diminish upon drug withdrawal. In contrast, accumulating evidence indicates that herbal medicines and plant-derived phytochemicals function as epigenetic reset signals capable of destabilizing entrenched epigenetic memory states rather than merely inhibiting individual regulatory nodes [6]. A defining feature of herbal medicines is their multi-component and multi-target nature, enabling simultaneous modulation of DNA methylation, histone modifications, and non-coding RNA networks. Numerous phytochemicals have been shown to downregulate DNMT1, DNMT3A, and DNMT3B expression while concomitantly restoring tumor-suppressive miRNAs that are epigenetically silenced in cancer cells [55,56]. When combined with miRNA-based interventions, such reset signals facilitate state transitions from memory-dominated epigenetic attractors toward reprogrammed cellular states characterized by reduced malignancy and enhanced therapeutic sensitivity. This framework reframes epigenetic intervention as a state transition process rather than transient gene suppression. This coordinated action directly perturbs DNMT–miRNA feedback loops, lowering the stability of bistable epigenetic states that otherwise lock cancer cells into resistant phenotypes. Importantly, herbal compounds rarely act as binary on–off switches. Instead, they impose graded, systems-level pressure on epigenetic networks by modulating redox balance, inflammatory signaling, and metabolic state–upstream regulators of DNMT activity and miRNA transcription [57,58]. Through sustained alteration of the intracellular redox and signaling landscape, herbal medicines can shift the regulatory thresholds governing DNMT–miRNA circuits, thereby facilitating transitions from memory-dominated states back toward a more plastic epigenetic configuration. Experimental studies support this reset model across diverse cancer contexts. Treatment with polyphenols, flavonoids, and traditional herbal extracts induce global DNA hypomethylation at tumor suppressor loci, re-expression of silenced miRNAs, and attenuation of cancer stem cell-associated transcriptional programs [55,59]. Notably, these effects often persist beyond the treatment window, consistent with partial erasure or reconfiguration of epigenetic memory rather than transient pathway inhibition. From a network perspective, herbal medicines are uniquely suited to disrupt epigenetic robustness. DNMT–miRNA feedback loops are embedded within larger regulatory architectures involving transcription factors, chromatin remodelers, and oxidative stress-responsive pathways. By targeting multiple layers of this architecture simultaneously, herbal medicines reduce redundancy and noise-buffering capacity, rendering epigenetic memory states more susceptible to collapse [60]. This property distinguishes herbal interventions from single-target epigenetic drugs, which may leave compensatory feedback loops intact. Within this framework, herbal medicine-induced miRNA modulation is conceptualized as a state-resetting input rather than a simple regulatory signal. By reactivating silenced miRNAs and weakening DNMT-mediated repression, herbal treatments promote shift from fixed attractor states toward transitional epigenetic landscapes that permit re-differentiation or therapeutic resensitization [57,58]. Such reprogramming capacity provides a mechanistic explanation for the long-observed phenomenon that herbal medicines often exhibit delayed yet durable anticancer effects. Collectively, herbal medicines function not merely as epigenetic modulators but as epigenetic reset signals that reconfigure DNMT–miRNA feedback networks, destabilize pathological epigenetic memory, and reopen phenotypic plasticity in cancer cells. Framing herbal interventions in this manner provides a conceptual bridge between traditional multi-component therapeutics and modern systems-level epigenetic reprogramming strategies. As illustrated in Figure 1, combined miRNA–herbal strategies act to destabilize DNMT–miRNA epigenetic memory circuits and facilitate durable phenotypic reprogramming.

### 2.5. Translational Implications of miRNA-Herbal Combination Strategies

Recognition of DNMT–miRNA feedback loops as epigenetic memory units provides a conceptual foundation for therapeutic strategies that move beyond transient gene modulation toward durable cellular reprogramming. Within this framework, the combination of miRNA-based interventions with herbal medicine emerges as compelling translational approach, as it simultaneously targets multiple regulatory layers stabilizing malignant cell states. Unlike single-agent epigenetic inhibitors, which often induce partial or reversible changes in DNA methylation, herbal medicines exert broad-spectrum regulatory effects intersecting with miRNA biogenesis, DNMT activity, chromatin accessibility, and redox signaling. Multiple studies demonstrate that herbal formulations and phytochemicals restore tumor-suppressive miRNA expression while attenuating DNMT1 or DNMT3A/3B activity, thereby weakening the self-reinforcing loops that sustain aberrant epigenetic states [61,62]. When combined with exogenous miRNA mimics or miRNA-inducing interventions, these effects synergistically destabilize entrenched epigenetic memory circuits. Mechanistically, miRNA–herbal combinations function as epigenetic reset signals rather than simple inhibitors. miRNAs directly disrupt core nodes of DNMT–miRNA feedback loops, while herbal compounds modulate upstream stress-responsive pathways—including ROS signaling, unfolded protein response, and inflammatory transcriptional programs—that initially and reinforce these loops. This dual targeting enables a transition from memory-dominated epigenetic states toward a reprogrammable, plastic phase in which cancer cells become susceptible to phenotypic reversal [63,64]. Preclinical evidence supports this model across multiple cancer contexts. Herbal formulations alter global and exosomal miRNA landscapes in vivo, reshaping tumor-associated epigenetic signatures and downstream signaling networks linked to proliferation, epithelial–mesenchymal transition, and drug resistance [65]. Importantly, these miRNA changes persist beyond the treatment window, consistent with partial rewriting of epigenetic memory rather than transient transcriptional suppression. Such durability is rarely achieved by conventional cytotoxic agents or pathway-specific inhibitors. Clinically, miRNA–herbal combination strategies offer several advantages. They provide a means to overcome therapy resistance rooted in epigenetic inertia, including resistance to chemotherapy, targeted therapy, and immunotherapy. The pleiotropic yet coordinated nature of herbal modulation reduces adaptive escape via single-pathway rewiring. Moreover, miRNA signatures induced by herbal treatment serve as dynamic biomarkers for monitoring epigenetic reprogramming and therapeutic response, enabling patient stratification and treatment optimization [66,67]. The translational relevance of this approach lies in its alignment with emerging concepts of precision epigenetic therapy. Rather than enforcing a fixed molecular target, miRNA–herbal combinations act on regulatory architectures—feedback loops, bistable switches, and chromatin memory modules—that define cancer cell identity. By targeting these architectures, such strategies aim not merely to eliminate cancer cells but to erode the epigenetic constraints locking them into malignant phenotypes. This perspective reframes herbal medicine from an adjunctive modality to a core component of RNA-based epigenetic reprogramming strategies in oncology [68,69]. Beyond mechanistic insight, translational studies demonstrate that herbal medicines and phytochemicals modulate miRNA expression and DNMT activity in a coordinated manner, resulting in durable epigenetic and phenotypic reprogramming. However, the translational scalability of miRNA–herbal combination strategies depends critically on rigorous phytochemical standardization supported by chromatographic fingerprinting and component-based quality systems [70,71], reproducible pharmacokinetic profiling across heterogeneous phytochemical classes [72,73,74], and mechanism-anchored PK/PD exposure–response integration to ensure network-level consistency [75]. Without quantitative fingerprinting and systems-level dose optimization, variability in bioactive composition may confound consistent DNMT–miRNA circuit modulation across clinical settings. Representative examples of herbal medicine-induced miRNA regulation, associated epigenetic targets, and translational implications are summarized in Table 2, illustrating how multi-component interventions function as epigenetic reset signals rather than transient pathway inhibitors.

### 2.6. Challenges, Limitations, and Clinical Feasibility of Epigenetic Reprogramming Strategies

Despite the conceptual appeal of miRNA–herbal combination strategies as epigenetic reset interventions, several translational challenges must be addressed before their broad clinical implementation. A primary limitation resides in the efficient, sustained, and tissue-specific delivery of miRNA-based therapeutics. Although advances in nanoparticle platforms and exosome-mediated delivery have improved miRNA stability and bioavailability, achieving durable and cell-type-selective modulation of miRNA networks in vivo remains a significant hurdle. A second critical challenge arises from the intrinsic heterogeneity of epigenetic states within tumors and across patients’ populations. DNMT–miRNA feedback architectures and their associated memory states vary according to tumor lineage, disease stage, and prior therapeutic exposure, implying that epigenetic reprogramming windows are highly context-dependent. Accordingly, biomarker-guided patient stratification will be essential to identify permissive reprogramming states and to avoid suboptimal or counterproductive interventions. Herbal medicines introduce additional layer of complexity due to variability in composition, bioactive content, and pharmacokinetics behavior. From a translational perspective, variability in phytochemical composition, bioavailability, metabolic transformation, and pharmacokinetic profiles may significantly influence therapeutic reproducibility and target engagement. Multi-component formulations often exhibit context-dependent pharmacodynamics, where synergistic or antagonistic interactions among constituents can alter DNMT–miRNA modulation in a dose- and tissue-specific manner. Therefore, rigorous chemical standardization, quantitative fingerprinting, pharmacokinetic–pharmacodynamic (PK–PD) modeling, and biomarker-guided dosing strategies will be essential to ensure clinical consistency and regulatory feasibility. Without such integrative validation frameworks, the therapeutic translation of herbal-based epigenetic modulation remains vulnerable to variability-driven efficacy and safety uncertainties. While multi-component formulations confer systems-level regulatory advantages, they also complicate standardization, dose optimization, and regulatory evaluation. Ensuring reproducibility, batch consistency, and mechanistic transparency will be therefore be essential for translating herbal-based epigenetic strategies into evidence-based clinical practice. Importantly, epigenetic reprogramming is not inherently benign. Excessive or prolonged disruption of epigenetic memory may induce unintended lineage plasticity, activation of compensatory survival pathways or destabilization of normal tissue identity. Mechanistically, epigenetic destabilization may inadvertently reactivate developmental transcriptional programs, induce stochastic chromatin remodeling, or promote partial EMT states that enhance cellular plasticity rather than enforce therapeutic differentiation. Moreover, global DNMT inhibition or redox-driven methylation turnover may affect non-target genomic loci, including tumor suppressor regions in normal tissues, thereby increasing the risk of off-target epigenomic drift. These risks underscore the necessity for locus-specific targeting strategies and temporally controlled modulation rather than indiscriminate epigenetic disruption. Defining optimal intervention intensity, duration, and sequencing will thus be critical to balance therapeutic benefit against epigenetic instability. From a clinical feasibility perspective, these limitations do not negate the potential of miRNA–herbal combination strategies but rather instead underscore the need for integrative and adaptive trial designs. Approaches incorporating longitudinal epigenetic profiling, circulating or exosomal miRNA biomarkers, and adaptive dosing frameworks may enable safe and effective translation of epigenetic reset concepts into clinical oncology. Importantly, although the concept of herbal medicines as epigenetic reset signals is mechanistically plausible, definitive longitudinal validation demonstrating stable methylome reconfiguration and durable DNMT–miRNA network rewiring in vivo remains limited. Most available studies rely on short-term molecular endpoints, and causal dissection of redox-dependent epigenetic modulation versus secondary transcriptional effects has not been comprehensively established. Future investigations integrating time-resolved methylome profiling, chromatin accessibility mapping, and mechanistic perturbation studies will be necessary to substantiate true epigenetic resetting rather than transient regulatory fluctuation. To ensure clarity regarding the strength of available data, the evidence discussed throughout this review should be interpreted according to its experimental hierarchy, including computational modeling, in vitro mechanistic studies, in vivo preclinical validation, and limited clinical observations. Computational and network-based predictions are considered hypothesis-generating frameworks unless supported by functional biological validation, and preclinical findings should not be equated with durable clinical efficacy. Explicit differentiation of these evidence levels is essential to avoid overinterpretation and to guide rational translational development. From a practical translational roadmap perspective, advancement of miRNA–herbal combination strategies may proceed through a sequential framework: (i) rigorous phytochemical standardization and quantitative fingerprinting to ensure compositional reproducibility; (ii) delivery optimization using validated platforms such as lipid nanoparticles or engineered exosomes for tissue-specific miRNA modulation; (iii) biomarker-guided patient stratification based on DNMT–miRNA network states or circulating/exosomal miRNA signatures; and (iv) adaptive dosing strategies incorporating longitudinal methylome and chromatin-state monitoring. Such a structured development pathway may reduce speculative interpretation and enhance the clinical feasibility of epigenetic reset interventions.

## 3. Conclusions and Future Perspectives

Recent advances in RNA biology and epigenetics have revealed that DNMT–miRNA feedback loops operate not merely as regulatory motifs but as epigenetic memory units that stabilize malignant cell states. This conceptual shift provides a coherent explanation for long-standing clinical observations—namely, why cancer phenotypes often persist after removal of initiating stimuli, why transient environmental or therapeutic pressures can induce durable cellular reprogramming, and why resistance frequently emerges despite effective target inhibition. Within this framework, miRNA-based interventions and herbal medicines converge on a shared therapeutic objective: destabilization of pathological epigenetic memory. miRNAs directly target the core nodes of DNMT–miRNA circuits, while herbal medicines impose systems-level modulation of upstream signaling, redox balance, and chromatin accessibility. When combined, these approaches function as coordinated epigenetic reset signals capable of reopening phenotypic plasticity rather than enforcing short-lived transcriptional suppression. This distinction reframes epigenetic therapy from enzyme inhibition toward state transition and identity reconfiguration. From a translational standpoint, this perspective supports a paradigm shift in oncology. Instead of relying solely on cytotoxic or pathway-specific agents, future therapeutic strategies may prioritize reprogramming tumor epigenetic landscapes to resensitize cancer cells to existing treatments or to constrain malignant evolution. miRNA–herbal combination strategies are particularly well suited to this goal, as their multi-target and adaptive nature aligns with the complex regulatory architectures that govern epigenetic memory. Looking ahead, several directions warrant focused investigation. First, the identification of epigenetic memory signatures—such as DNMT–miRNA network states or exosomal miRNA profiles—could enable patient stratification and dynamic monitoring of reprogramming efficacy. Second, optimized sequencing and dosing strategies will be required to balance effective memory destabilization with epigenetic safety. Third, integration of systems biology modeling with longitudinal clinical data may refine the timing and context in which epigenetic reset interventions are most effective. In summary, viewing DNMT–miRNA interactions through the lens of epigenetic memory unifies mechanistic insight with translational opportunity. miRNA–herbal combination strategies exemplify how traditional multi-component therapeutics and modern RNA-based approaches can be integrated into a coherent, forward-looking model of precision epigenetic medicine. By targeting regulatory architectures rather than isolated molecular nodes, such strategies hold promises for overcoming therapeutic resistance and achieving durable clinical benefit in cancer.

## Figures and Tables

**Table 1:** DNMT–miRNA feedback loops as epigenetic memory units across cancer types.

Cancer Type	miRNA(s) Involved	DNMT Target(s)	Feedback Loop Characteristic	Epigenetic/Phenotypic Outcome	Ref.
Gastric cancer	miR-200c	DNMT3A	Double-negative feedback	Stable EMT state, invasive phenotype	[40]
Lung cancer (cisplatin-resistant)	miR-30a/c	DNMT1	Self-reinforcing loop	Persistent drug resistance	[45]
Colorectal cancer	miR-148a	DNMT1	Bistable regulation	DNA hypermethylation, stemness	[41]
Breast cancer	miR-29 family	DNMT3A/3B	Reciprocal repression	Long-term silencing of tumor suppressors	[42]
Hepatocellular carcinoma	miR-152	DNMT1	Memory-like feedback	Epigenetic maintenance of malignancy	[46]
Multiple cancers (modeling)	Oltipraz	DNMT network	Bistable/attractor-based	Noise-resistant epigenetic memory	[25]

Feedback loop characteristics are categorized based on experimentally validated mechanistic studies where available; in cases lacking direct perturbation evidence, interactions are described as inferred from correlative or modeling-based analyses. Abbreviations: DNMT, DNA methyltransferase; miRNA, microRNA; EMT, epithelial–mesenchymal transition; HCC, hepatocellular carcinoma.

**Table 2:** Herbal medicine-induced miRNA modulation and epigenetic reprogramming in cancer models.

Herbal Medicine/Compound	Cancer Model	miRNA(s) Regulated	Epigenetic Target(s)	Functional Outcome	Translational Implication	Ref.
Curcumin	Breast, colon	miR-21 ↓, miR-200 ↑	DNMT1 ↓	EMT reversal, apoptosis	Epigenetic resensitization	[56]
Resveratrol	Prostate, lung	miR-34a ↑	DNMT3B ↓	Growth inhibition	Combination with miRNA therapy	[76]
EGCG	Colorectal	miR-16 ↑	DNMT1 ↓, Global hypomethylation	Tumor suppressor reactivation	Memory destabilization	[55]
Berberine	HCC	miR-152 ↑	DNMT1 ↓	Reduced invasion	Epigenetic reset signal	[57]
Multi-herbal formula	Various	Exosomal miRNAs ↑	DNMT network modulation	Long-lasting phenotypic change	Biomarker-guided therapy	[59]
Herbal + miRNA mimic	Preclinical models	Targeted miRNAs	DNMT–miRNA loop (preclinical)	Durable epigenetic modulation	Translational combinatorial strategy	[63]

Feedback loop characteristics are categorized based on experimentally validated mechanistic studies where available; in cases lacking direct perturbation evidence, interactions are described as inferred from correlative or modeling-based analyses. Abbreviations: ↓ downregulation; ↑ upregulation; DNMT, DNA methyltransferase; miRNA, microRNA; EMT, epithelial–mesenchymal transition; EGCG, epigallocatechin gallate; HCC, hepatocellular carcinoma.

## Data Availability

No new data were created or analyzed in this study. Data sharing is not applicable to this article.
